# Left-Sided Heart Defects and Laterality Disturbance in Hypoplastic Left Heart Syndrome

**DOI:** 10.3390/jcdd10030099

**Published:** 2023-02-24

**Authors:** Hisato Yagi, Cecilia W. Lo

**Affiliations:** Department of Developmental Biology, School of Medicine, University of Pittsburgh, Pittsburgh, PA 15201, USA

**Keywords:** left–right patterning, laterality, hypoplastic left heart syndrome, Sin3A, heterotaxy

## Abstract

Hypoplastic left heart syndrome (HLHS) is a complex congenital heart disease characterized by hypoplasia of left-sided heart structures. The developmental basis for restriction of defects to the left side of the heart in HLHS remains unexplained. The observed clinical co-occurrence of rare organ situs defects such as biliary atresia, gut malrotation, or heterotaxy with HLHS would suggest possible laterality disturbance. Consistent with this, pathogenic variants in genes regulating left–right patterning have been observed in HLHS patients. Additionally, *Ohia* HLHS mutant mice show splenic defects, a phenotype associated with heterotaxy, and HLHS in *Ohia* mice arises in part from mutation in *Sap130*, a component of the Sin3A chromatin complex known to regulate *Lefty1* and *Snai1*, genes essential for left–right patterning. Together, these findings point to laterality disturbance mediating the left-sided heart defects associated with HLHS. As laterality disturbance is also observed for other CHD, this suggests that heart development integration with left–right patterning may help to establish the left–right asymmetry of the cardiovascular system essential for efficient blood oxygenation.

## 1. Introduction

Hypoplastic left heart syndrome (HLHS) is a congenital heart disease (CHD) with severe hypoplasia of left-sided heart structures, including hypoplasia of the aorta (Ao), aortic and mitral valves (MV), and the left ventricle (LV). While HLHS infants now survive their critical CHD [1,2] with a three-stage surgical course that creates a “single ventricle” (SV) physiology with the right ventricle (RV) serving as the systemic pumping chamber, there is nevertheless developmental delay, poor growth, high risk of heart failure, and impaired neurodevelopment [3,4,5,6,7,8,9,10]. The 10-year transplant-free survival is only 39–50% [11,12,13], with the greatest risk seen in the first year of life with 30% mortality reported [14,15,16]. In addition, a host of clinical complications are observed after the third stage Fontan procedure in adult HLHS survivors. This can include heart failure with need for heart transplantation, liver cirrhosis, hepatocellular carcinoma, protein losing enteropathy, renal failure, and other clinical complications [17]. Hence, substantive improvement in outcome for HLHS patients will likely require interventions to recover LV growth. With enhanced LV growth, a two-ventricle surgical path may be possible, which can avoid the adverse clinical sequelae associated with an SV physiology. This will require in utero intervention, as cardiomyocyte proliferation largely ceases postnatally, but without mechanistic insights into why the ventricular hypoplasia is left-sided, no therapy is available to restore LV growth in fetuses with HLHS.

## 2. Co-Occurrences of Laterality Defects with HLHS

HLHS has been reported to co-occur with various laterality defects, indicating a possible role for left–right patterning disturbances in the restriction of defects to the left side of the heart [18]. Thus, HLHS has been reported in subjects with heterotaxy, situs inversus, gut malrotation [19,20], biliary atresia [21], or spleen abnormalities [20]. This has been observed not only in case reports, but also in studies involving systematic analysis of subjects in different birth defects registries [18,22,23]. In one such study, data from the National Birth Defects Prevention Study (NBDPS) was used to examine for CHD in 517 nonsyndromic laterality defect cases. This showed that 3.2% of the subjects with laterality defects also had HLHS (Table 1). Conversely, another study conducted on 12,000 infants with CHD examined for co-occurrence with other birth defects including laterality defects [23]. This analysis revealed 4.7% of HLHS subjects had situs inversus totalis, 6.5% had gut malrotation, and 7.7% had spleen abnormalities (Table 1). As HLHS is a relatively rare CHD lesion with incidence of 1–5% among complex CHD, and heterotaxy and laterality phenotypes have an incidence of 1 in 10,000 [24], their co-occurrence is unlikely to occur by chance. Similarly, biliary atresia, which is reported at an incidence of 1 in 8000 to 18,000 [25,26,27,28,29], also can be seen with HLHS. In line with findings from these large birth registry studies, another recent study of 70 patients with laterality and cardiac defects revealed 40% had single ventricle heart defects [30]. Overall, these findings support a mechanistic link between HLHS and the disturbance of laterality.

## 3. Association of HLHS with Genes Involved in the Specification of Left–Right Asymmetry

HLHS in conjunction with other closely related left ventricular outflow tract obstructive (LVOTO) CHD lesions have shown high heritability, providing strong evidence of a genetic etiology for HLHS [31,32,33,34,35]. In this regard, it is worth noting that several genes known to cause heterotaxy, such as *ZIC3* [36], *FGFR4* [37], and *FOXF1* associated with bowel malrotation [19], are associated with HLHS. This would support a shared genetic etiology for HLHS and heterotaxy. Also notable is the finding of mutations in *HAND1* associated with HLHS [38,39]. *HAND1* encodes a transcription factor exhibiting restricted expression in the embryonic left ventricle, indicating an essential role in formation of the LV. However, mutant mice with a deficiency in these laterality genes have not replicated the HLHS phenotype, nor have knockin of a A126FS *HAND1* somatic mutation recovered from HLHS heart tissue. In the latter mutant mice, heart outflow tract defects and intraventricular abnormalities were observed, but the LV was of normal size [40].

From a large-scale mouse mutagenesis screen for mutations causing CHD, we recovered eight independent mouse lines with HLHS, including one mutant line named *Ohia* in which the HLHS causing mutations were identified. This was shown to be mediated by two genes—a point mutation in Protocadherin A9 (*Pcdha9*) and a splicing defect mutation in Sin3A associated protein 130 (*Sap130*). Mice with CRISPR gene edited alleles of *Pcdha9/Sap130* replicated the HLHS phenotype, confirming their role in the pathogenesis of HLHS seen in the *Ohia* mutant line. While the *Pcdha9* mutation was shown to cause the aorta/aortic valve defects in HLHS, the *Sap130* mutation was shown to play a pivotal role in the LV hypoplasia [41]. The fact that *Sap130* also may contribute to the defects being left-sided is supported by observations related to the function of the Sin3A complex (see below).

The cardiac phenotype in the *Ohia* mutant line spanned a spectrum, with 26% exhibiting HLHS among mice that are double homozygous for the *Sap130/Pcdha9* mutations [41]. Of the remaining, 7% exhibited LV/mitral valve hypoplasia but with a normal sized aorta, 16% had an isolated hypoplastic aorta, and 34% exhibited other CHD. Among the HLHS mutants analyzed (n = 28), five (18%) exhibited a double outlet right ventricle (DORV) variant subtype of HLHS. Such incomplete penetrance and variable expressivity are reminiscent of what is often observed in familial studies of CHD. This is thought to reflect the genetic heterogeneity of the human population, but as our studies are conducted entirely in inbred mice, with similar results seen in the CRISPR gene edited *Ohia* mutant line, the incomplete penetrance and variable expressivity must have an epigenetic etiology that likely reflects gene–environment interactions.

**Figure 1:**
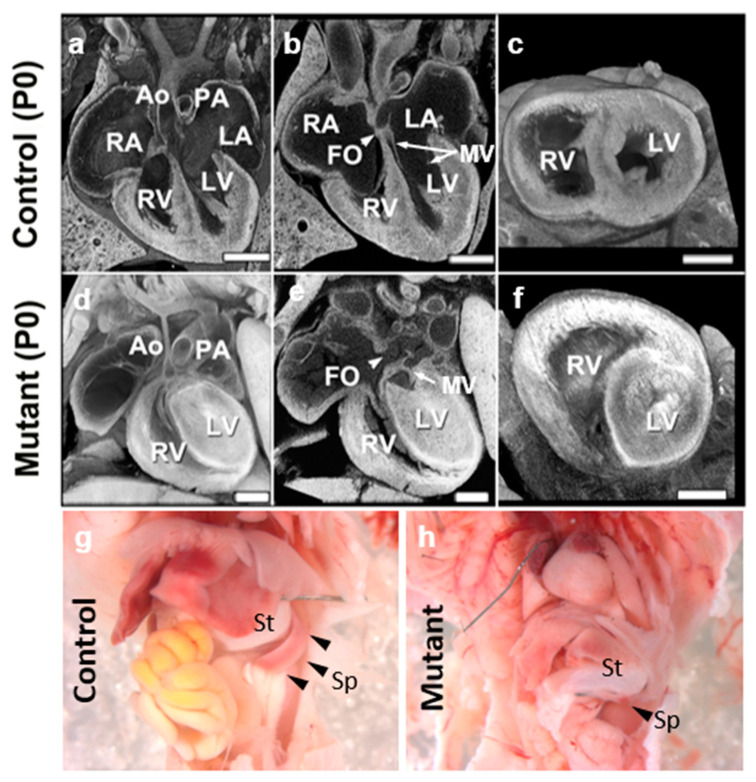
*Ohia* HLHS mutant mice exhibit left heart and spleen hypoplasia. (**a**–**f**) Histopathology showing the cardiac anatomy of HLHS mutant mouse and littermate control at birth (P0). Note the muscle-bound LV with no lumen, hypoplastic aorta, aortic valve atresia, and mitral valve (MV) hypoplasia/stenosis, and patent foramen ovale (FO) denoted by white arrowhead. The interventricular septum is intact in the mutant mouse. (**g**,**h**) Newborn wildtype (**g**) and *Ohia* mutant (**h**) mice. Spleen (black arrowheads in (**g**,**h**)) hypoplasia is observed in the *Ohia* mutant as compared to the wildtype (see black arrowhead) mouse. LA, left atrium; RA, right atrium; LV, left ventricle; RV, right ventricle, MV: mitral valve, St; Stomach, Sp; Spleen. Scale bars: 0.5 mm.

It is also interesting to note that the *Ohia* mutant mice can have a hypoplastic spleen or asplenia (Figure 1g,h), defects also seen with heterotaxy, supporting involvement of laterality disturbance in HLHS. As the spleen is found ventroposterior to the stomach, which is positioned on the left-side of the body, the spleen is considered a left-sided structure. Thus, hypoplastic spleen or asplenia in *Ohia* mutant mice would constitute a left-sided defect, in line with hypoplasia of left-sided heart structures in HLHS. This would suggest possible right isomerism. In contrast, we note clinically that HLHS has been suggested to involve left isomerism, as azygos or hemiazygos venous connection can be seen in HLHS patients. However, as azygous venous connection can occur in the context of normal atrial situs, identifying atrial situs via venous connections alone can also lead to erroneous diagnosis of left isomerism. In addition, as right atrial isomerism is one of the most severe forms of CHD, and is associated with high morbidity and mortality, right isomerism may be underrepresented in the viable HLHS patient population, resulting in ascertainment bias of left-isomerism [42]. We note neither azygous continuation nor atrial situs anomalies have been observed in the *Ohia* HLHS mouse model.

## 4. Hippo Signaling and LV Hypoplasia in HLHS

While severe hypoplasia of the LV is a major hallmark of HLHS, the hypoplasia is observed not only in the LV, but in all the structures on the left side of the heart, including the left atrium, aorta, and aortic and mitral valves. This suggests disturbance of heart organ size regulation, albeit restricted to the left side. Relevant to this, Hippo signaling has been shown to regulate heart organ size with the modulation of cell proliferation, apoptosis, and stem cell self-renewal [43]. This involves a kinase cascade that regulates nuclear trafficking of the YAP transcription factor such that when the Hippo pathway is on, a protein phosphorylation cascade is activated promoting nuclear exit and cytoplasmic degradation of the transcriptional coactivators YAP and TAZ. In contrast, when Hippo pathway is off, YAP/TAZ are dephosphorylated, and translocated into the nucleus where they form a complex with TEAD co-transcriptional regulators to activate the transcription of genes regulating cell proliferation and stem cell renewal.

Genetic manipulation of Hippo-Yap signaling in mice has shown an essential role for the Hippo pathway in heart organ size regulation. This involves the inhibition of cardiomyocyte proliferation via repression of Wnt target genes [44]. Reduction in Hippo signaling has also been shown to allow myocardial regeneration by promoting adult cardiomyocytes to re-enter the cell cycle [45]. Hippo-deficiency in cardiomyocytes in a mouse infarct model was observed to reduce infarct scar size while increasing survival, suggesting the restoration of cardiomyocyte proliferative potential [45]. Moreover, cardiomyocyte proliferation was observed in the adult heart with Hippo deficiency in an apical ventricular resection injury model [45]. Significantly, a role for Hippo signaling in regulating cardiomyocyte proliferation in HLHS was recently demonstrated with the observation that iPSC-CM from HLHS patients exhibited loss of YAP nuclear localization [46]. This was accompanied by reduced cardiomyocyte proliferation and increased apoptosis. However, upon restoration of YAP nuclear localization, proliferation of the HLHS iPSC-CM was also restored [46]. These findings support the essential role of altered Hippo signaling in the LV hypoplasia in HLHS, but why the defects are restricted to the left side of the heart remains unexplained by these observations.

We note a recent study which suggested that Hippo signaling does not play a role in normal organ growth. However, this study investigated only the mouse liver and used conditional deletion of Yap/Taz or hyperactivation with YAP overexpression or Lats1/2 conditional deletion [47]. In contrast, we recently showed in analysis of HLHS patient iPSC-cardiomyocytes, even as YAP transcript and protein expression were unchanged, that there was a marked YAP protein trafficking defect. This would suggest that the role of Hippo signaling in organ growth may involve more subtle regulation that cannot be elucidated with loss or gain of function studies. Additionally, even as Hippo signaling is said to regulate abnormal ectopic growth, abnormal Hippo signaling could also contribute to the abnormal LV hypoplasia in HLHS, regardless of its role in normal organ growth. More studies are needed to examine both the role of Hippo signaling in normal organ size regulation and the impact of dysregulated YAP protein trafficking in the LV hypoplasia in HLHS.

## 5. Sap130/Sin3A in Heart Organ Size Regulation and Left–Right Patterning in HLHS

Sap130 is a component of the Sin3A-HDAC repressor complex, a chromatin modifying complex with critical roles in a wide spectrum of developmental processes such as the maintenance of stem cell pools [48], muscle differentiation [49], and the regulation of neuronal differentiation/neurodevelopment in conjunction with REST/CoREST [50]. Sin3A deficiency can cause Witteveen–Kolk syndrome, a neurodevelopmental disorder with intellectual disability, ADHD, autism, and other defects including microcephaly [51], with phenotypes overlapping those in HLHS patients. Sap130 chromatin immunoprecipitation sequencing (ChIPseq) showed chromatin occupancy that indicated a role for Sap130 in the regulation of genes in the Hippo-YAP pathway, a pathway previously shown to regulate organ size, including the heart [43]. Thus, Sap130 ChIPseq analysis of the embryonic heart recovered YAP, the Hippo kinases, and the TEAD family of transcriptional factors.

Consistent with a role in heart organ size regulation, Yap1 has been shown to regulate cardiomyocyte proliferation from the very earliest stages of mouse heart development, with Yap1 and Tead observed to be strongly expressed and nuclear localized in the E8.5 myocardium [52]. The *Nkx2.5*-Cre mediated cardiac specific deletion of *Yap1* resulted in embryos with smaller hearts, evident by E9.5 [53]. This was associated with a 50% reduction in the number of cardiomyocytes and a significant reduction in proliferating cells in the heart [53]. These *Nkx2.5*-Cre *Yap1* deleted embryos died by E10.5 from heart failure with a thin myocardium. In a separate study, *Nkx2.5*-Cre deletion of a floxed *Sav1* allele, a Hippo kinase in the Mst1/Mst2 complex, resulted in cardiomegaly due to failure to repress cardiomyocyte proliferation normally regulated by Hippo signaling. As the heart enlargement affected the RV and LV equally, this indicated that Hippo regulation of ventricular growth has no intrinsic “sidedness” [44]. This would suggest that *Sap130* likely plays a role in specifying the sidedness of the ventricular hypoplasia in HLHS. This may involve *Sap130/Sin3A*-mediated integration of pathways regulating left–right patterning with Hippo signaling and the regulation of cardiomyocyte proliferation.

**Figure 2:**
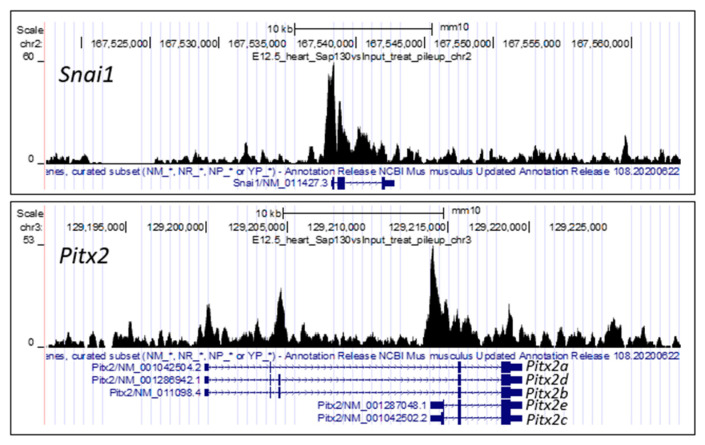
Sap130 ChIP–seq of E12.5 mouse heart. Sap130 antibody was used for chromatin immunoprecipitation sequencing, identifying Sap130 occupancy over the promoter regions of left–right patterning genes *Snai1* and *Pitx2*. It should be noted fir *Pitx2*, transcripts for three isoforms are shown, *Pitx2a*, *b*, *c*, *d*, *e. Pitx2c* is the isoform associated with left–right patterning.

This is supported by the observation that two genes with essential functions in left–right patterning, *Lefty1* and *Snai1*, are both found in the Sin3A complex. Sin3A has been shown to recruit the DNA demethylase Tet1 to demethylate the promoter of *Lefty1* and thus enhance its transcription [54]. Expression of *Lefty1* is required for the breaking of symmetry and the specification of left-sidedness. In addition, Snai1 is also found in the Sin3A complex mediating the transcriptional repression of cadherins [55] to promote epithelial–mesenchymal transition, a developmental process critical for left–right patterning [56]. Thus, *Snai1* knockout mice exhibit heterotaxy with disruption of left–right patterning [57]. ChIPseq analysis of E12.5/E14.5 embryonic heart tissue showed that *Snai1* and *Pitx2c* are likely Sap130 targets (Figure 2); *Pitx2c* being a transcription factor specifying left-sided visceral organ identity [58,59]. Both genes are expressed in the mouse heart at these stages, while Lefty1, which is not expressed at these late stages of heart development, was not observed by Sap130 ChIPseq analysis. These findings suggest that Sap130 specifically recruits transcriptional regulators to the Sin3A complex to regulate transcriptional programming related to growth and development of left-sided heart structures.

## 6. Left–Right Patterning Integral to Heart Development and the Pathogenesis of CHD

The overall evidence in studies of patients, mice, and other animal models would suggest the involvement of laterality disturbance in the developmental etiology of HLHS. As none of the genes or variants recovered from HLHS patients known to regulate left–right patterning yielded HLHS or a small LV phenotype in KO/KI mouse models, this would indicate that these genes are likely functioning as genetic modifiers to bias the phenotype towards left-sided heart structures. This fits well with several studies indicating an oligogenic etiology for HLHS [33,41]. Such an oligogenic model of disease involving the disturbance of left–right patterning may also have relevance for other CHD lesions. Thus, we noted a previous study which showed *Smad2/Nodal* double heterozygous KO mice exhibiting laterality defects in conjunction with CHD, comprising transposition of the great artery [60].

Of further note, studies conducted using birth registries have also shown a wide spectrum of other CHD associated with laterality defects (Table 1). Thus, situs anomalies were seen in 5–14% of atrioventricular septal defects (AVSD), total anomalous pulmonary venous return (TAPVR), and double outlet right ventricle (DORV) (Table 1), with an even higher incidence seen in SV lesions, with 17.6% for situs inversus, 11.8% for gut malrotation, and 23.5% for splenic abnormalities (Table 1). These findings suggest heart development is tightly integrated with left–right patterning, and thus disturbance of one can often affect the other. We note anecdotal reports of the association of various birth defects typically seen with heterotaxy, such as that azygos or hemiazygos venous connection of the inferior vena cava or biliary atresia have been reported in case studies of patients with HLHS. In one study of 163 heterotaxy patients with left atrial isomerism, 10% biliary atresia and 8% hypoplastic LV were reported [61], but the association of biliary atresia with hypoplastic LV may not to represent classic HLHS [21].

## 7. Summary and Clinical Implications

Establishing the critical left–right asymmetry in the cardiovascular system is an evolutionary adaption essential for the separation of oxygenated from deoxygenated blood to allow efficient blood oxygenation in air-breathing animals. This integration of heart development with left–right patterning may account for the cardiovascular system being the most left–right asymmetric organ in the mammalian body plan. Thus, understanding how left–right patterning is integrated with cardiac morphogenesis may provide important insights not only into CHD such as HLHS, but likely a broad spectrum of other CHD beyond HLHS. Ultimately, insights into how left–right patterning is integrated with heart development may help to identify new druggable targets for the development of therapeutics for recovering LV growth in fetuses with evolving HLHS. This may provide a two-ventricle surgical path that holds promise for better long-term outcomes for patients otherwise diagnosed with HLHS.

## Figures and Tables

**Table 1:** Prevalence of Laterality Defects Among Different Types of Congenital Heart Defects *.

	HLHS	SV	TA	PTA	PA	TGA	DORV	AVSD/ ECD	TAPVR	TOF	EB	Total
**A. CHD Associated with Heterotaxy and Situs Inversus in National Birth Defects Prevention Study by Lin et al.** [22]												
**Situs Inversus**	1.4%	5.8%	0.7%	0%	1.4%	2.4%	7.9%	5.0%	2.2%	2.9%	0.7%	43.2%
**Heterotaxy**	3.2%	14.0%	2.1%	1.1%	9.8%	3.7%	25.7%	48.4%	31.2%	4.2%	0.3%	96.6%
**All Laterality**	2.7%	11.8%	1.7%	0.8%	4.9%	3.1%	20.9%	36.8%	23.4%	3.9%	0.4%	82.2%
**B. Situs Anomalies Associated with CHD in Birth Defects Registry Study on Congenital Malformations by Pradat et al.** [23]												
**Situs Inversus**	4.7%	17.6%	0%	1.0%	9.8%	5.7%	13.6%	9.4%	4.8%	3.7%	5.0%	5.6%
**Gut Malrotation**	6.5%	11.8%	0%	7.1%	4.9%	2.4%	11.9%	10.4%	6.3%	4.9%	0%	6.2%
**Spleen Abnormal**	7.7%	23.5%	4.1%	6.1%	4.9%	4.9%	8.5%	11.3%	12.7%	5.8%	0%	7.5%

* HLHS, hypoplastic left heart syndrome; SV, single ventricle; TA, tricuspid atresia; PTA, persistent truncus arteriosus; PA, pulmonary atresia; TGA, transposition of the great arteries; DORV, double outlet right ventricle; AVSD/ECD, atrioventricular septal defect/endocardial cushion defect; TAPVR, total anomalous pulmonary venous return; TOF, tetralogy of Fallot; EB, Ebstein’s anomaly.

## Data Availability

The mouse data that support the finding of this study are openly available in [41] at doi.org/10.1038/ng.3870, and are available from the corresponding author on reasonable request.

## References

[1] Siffel C., Riehle-Colarusso T., Oster M.E., Correa A. (2015). Survival of Children With Hypoplastic Left Heart Syndrome. Pediatrics.

[2] Yabrodi M., Mastropietro C.W. (2016). Hypoplastic left heart syndrome: From comfort care to long-term survival. Pediatr. Res..

[3] Burch P.T., Gerstenberger E., Ravishankar C., Hehir D.A., Davies R.R., Colan S.D., Sleeper L.A., Newburger J.W., Clabby M.L., Williams I.A. (2014). Longitudinal Assessment of Growth in Hypoplastic Left Heart Syndrome: Results From the Single Ventricle Reconstruction Trial. J. Am. Heart Assoc..

[4] Goldberg C.S., Mussatto K., Licht D., Wernovsky G. (2011). Neurodevelopment and quality of life for children with hypoplastic left heart syndrome: Current knowns and unknowns. Cardiol. Young.

[5] Donofrio M.T., Duplessis A.J., Limperopoulos C. (2011). Impact of congenital heart disease on fetal brain development and injury. Curr. Opin. Pediatr..

[6] Morton P.D., Ishibashi N., Jonas R.A. (2017). Neurodevelopmental Abnormalities and Congenital Heart Disease. Circ. Res..

[7] Volpe J.J. (2014). Encephalopathy of Congenital Heart Disease– Destructive and Developmental Effects Intertwined. J. Pediatr..

[8] Newburger J.W., Sleeper L.A., Frommelt P.C., Pearson G.D., Mahle W.T., Chen S., Dunbar-Masterson C., Mital S., Williams I.A., Ghanayem N.S. (2014). Transplantation-Free Survival and Interventions at 3 Years in the Single Ventricle Reconstruction Trial. Circulation.

[9] Hoang T.T., Goldmuntz E., Roberts A.E., Chung W.K., Kline J.K., Deanfield J.E., Giardini A., Aleman A., Gelb B.D., Mac Neal M. (2018). The Congenital Heart Disease Genetic Network Study: Cohort description. PLoS ONE.

[10] Ravishankar C., Zak V., Williams I.A., Bellinger D.C., Gaynor J.W., Ghanayem N.S., Krawczeski C.D., Licht D.J., Mahony L., Newburger J.W. (2012). Association of Impaired Linear Growth and Worse Neurodevelopmental Outcome in Infants with Single Ventricle Physiology: A Report from the Pediatric Heart Network Infant Single Ventricle Trial. J. Pediatr..

[11] Driscoll D.J., Offord K.P., Feldt R.H., Schaff H.V., Puga F.J., Danielson G.K. (1992). Five- to fifteen-year follow-up after Fontan operation. Circulation.

[12] Gentles T.L., Gauvreau K., Mayer J.E., Fishberger S.B., Burnetta J., Colan S.D., Newburger J.W., Wernovsky G. (1997). Functional outcome after the Fontan operation: Factors influencing late morbidity. J. Thorac. Cardiovasc. Surg..

[13] Garcia M., Firek M., Zakhary B., Brenner M., Hildebrand F., Coimbra R. (2020). Severe Pelvic Fracture in the Elderly: High Morbidity, Mortality, and Resource Utilization. Am. Surg..

[14] Tweddell J.S., Hoffman G.M., Mussatto K.A., Fedderly R.T., Berger S., Jaquiss R.D.B., Ghanayem N.S., Frisbee S.J., Litwin S.B. (2002). Improved Survival of Patients Undergoing Palliation of Hypoplastic Left Heart Syndrome: Lessons Learned From 115 Consecutive Patients. Circulation.

[15] Alsoufi B., Mahle W.T., Manlhiot C., Deshpande S., Kogon B., McCrindle B.W., Kanter K. (2015). Outcomes of heart transplantation in children with hypoplastic left heart syndrome previously palliated with the Norwood procedure. J. Thorac. Cardiovasc. Surg..

[16] Cleves M.A., Ghaffar S., Zhao W., Mosley B.S., Hobbs C.A. (2003). First-year survival of infants born with congenital heart defects in Arkansas (1993-1998): A survival analysis using registry data. Birth Defects Res. Part A Clin. Mol. Teratol..

[17] Wilson W.M., Valente A.M., Hickey E.J., Clift P., Burchill L.J., Emmanuel Y., Gibson P., Greutmann M., Grewal J., Grigg L.E. (2018). Outcomes of Patients With Hypoplastic Left Heart Syndrome Reaching Adulthood After Fontan Palliation. Circulation.

[18] Gabriel G.C., Lo C.W. (2020). Left–right patterning in congenital heart disease beyond heterotaxy. Am. J. Med. Genet. Part C Semin. Med. Genet..

[19] Stankiewicz P., Sen P., Bhatt S.S., Storer M., Xia Z., Bejjani B.A., Ou Z., Wiszniewska J., Driscoll D.J., Bolivar J. (2009). Genomic and Genic Deletions of the FOX Gene Cluster on 16q24.1 and Inactivating Mutations of FOXF1 Cause Alveolar Capillary Dysplasia and Other Malformations. Am. J. Hum. Genet..

[20] Huseynova R.A., Bin Mahmoud L.A., Abdelrahim A., Alroiedy M.A., Huseynov O. (2020). Polysplenia syndrome with complex heart disease and jejunal atresia with malrotation in neonate: A case report. Clin. Case Rep..

[21] Becker D.J., Islam S., Geiger J.D. (2004). Biliary atresia associated with hypoplastic left heart syndrome: A case report and review of the literature. J. Pediatr. Surg..

[22] Lin A.E., Krikov S., Riehle-Colarusso T., Frías J.L., Belmont J., Anderka M., Geva T., Getz K.D., Botto L.D., The National Birth Defects Prevention Study (2014). Laterality defects in the national birth defects prevention study (1998-2007): Birth prevalence and descriptive epidemiology. Am. J. Med. Genet. Part A.

[23] Pradat P., Francannet C., Harris J., Robert E. (2003). The Epidemiology of Cardiovascular Defects, Part I: A Study Based on Data from Three Large Registries of Congenital Malformations. Pediatr. Cardiol..

[24] Peeters H., Devriendt K. (2006). Human laterality disorders. Eur. J. Med. Genet..

[25] Narkewicz M.R. (2001). Biliary atresia: An update on our understanding of the disorder. Curr. Opin. Pediatr..

[26] Perlmutter D.H., Shepherd R.W. (2002). Extrahepatic biliary atresia: A disease or a phenotype?. Hepatology.

[27] Schreiber R.A., Kleinman R.E. (2002). Biliary Atresia. J. Craniofacial Surg..

[28] Lupo P.J., Isenburg J.L., Salemi J.L., Mai C.T., Liberman R.F., Canfield M.A., Copeland G., Haight S., Harpavat S., Hoyt A.T. (2017). Population-based birth defects data in the United States, 2010-2014: A focus on gastrointestinal defects. Birth Defects Res..

[29] Bates M.D., Bucuvalas J.C., Alonso M.H., Ryckman F.C. (1998). Biliary Atresia: Pathogenesis and Treatment. Semin. Liver Dis..

[30] Liu S., Wei W., Wang P., Liu C., Jiang X., Li T., Li F., Wu Y., Chen S., Sun K. (2022). LOF variants identifying candidate genes of laterality defects patients with congenital heart disease. PLoS Genet..

[31] McBride K.L., Zender G.A., Fitzgerald-Butt S.M., Koehler D., Menesses-Diaz A., Fernbach S., Lee K., Towbin J.A., Leal S., Belmont J.W. (2009). Linkage analysis of left ventricular outflow tract malformations (aortic valve stenosis, coarctation of the aorta, and hypoplastic left heart syndrome). Eur. J. Hum. Genet..

[32] Hinton R.B., Martin L.J., Rame-Gowda S., Tabangin M.E., Cripe L.H., Benson D.W. (2009). Hypoplastic Left Heart Syndrome Links to Chromosomes 10q and 6q and Is Genetically Related to Bicuspid Aortic Valve. J. Am. Coll. Cardiol..

[33] McBride K., Pignatelli R., Lewin M., Ho T., Fernbach S., Menesses A., Lam W., Leal S.M., Kaplan N., Schliekelman P. (2005). Inheritance analysis of congenital left ventricular outflow tract obstruction malformations: Segregation, multiplex relative risk, and heritability. Am. J. Med. Genet. Part A.

[34] Hinton R.B., Martin L.J., Tabangin M.E., Mazwi M.L., Cripe L.H., Benson D.W. (2007). Hypoplastic Left Heart Syndrome Is Heritable. J. Am. Coll. Cardiol..

[35] Benson D.W., Martin L.J., Lo C.W. (2016). Genetics of Hypoplastic Left Heart Syndrome. J. Pediatr..

[36] Cowan J., Tariq M., Ware S.M. (2013). Genetic and Functional Analyses of *ZIC3* Variants in Congenital Heart Disease. Hum. Mutat..

[37] Sempou E., Lakhani O.A., Amalraj S., Khokha M.K. (2018). Candidate Heterotaxy Gene FGFR4 Is Essential for Patterning of the Left-Right Organizer in Xenopus. Front. Physiol..

[38] Hickey E.J., Caldarone C.A., McCrindle B.W. (2012). Left Ventricular Hypoplasia: A Spectrum of Disease Involving the Left Ventricular Outflow Tract, Aortic Valve, and Aorta. J. Am. Coll. Cardiol..

[39] Reamon-Buettner S.M., Ciribilli Y., Traverso I., Kuhls B., Inga A., Borlak J. (2009). A functional genetic study identifies HAND1 mutations in septation defects of the human heart. Hum. Mol. Genet..

[40] Firulli B.A., Toolan K.P., Harkin J., Millar H., Pineda S., Firulli A.B. (2017). The HAND1 frameshift A126FS mutation does not cause hypoplastic left heart syndrome in mice. Cardiovasc. Res..

[41] Liu X., Yagi H., Saeed S., Bais A.S., Gabriel G.C., Chen Z., Peterson K.A., Li Y., Schwartz M.C., Reynolds W.T. (2017). The complex genetics of hypoplastic left heart syndrome. Nat. Genet..

[42] Shiraishi I., Ichikawa H. (2012). Human Heterotaxy Syndrome. Circ. J..

[43] Wang J., Liu S., Heallen T., Martin J.F. (2018). The Hippo pathway in the heart: Pivotal roles in development, disease, and regeneration. Nat. Rev. Cardiol..

[44] Heallen T., Zhang M., Wang J., Bonilla-Claudio M., Klysik E., Johnson R.L., Martin J.F. (2011). Hippo Pathway Inhibits Wnt Signaling to Restrain Cardiomyocyte Proliferation and Heart Size. Science.

[45] Heallen T., Morikawa Y., Leach J., Tao G., Willerson J.T., Johnson R.L., Martin J.F. (2013). Hippo signaling impedes adult heart regeneration. Development.

[46] Xu X., Jin K., Bais A.S., Zhu W., Yagi H., Feinstein T.N., Nguyen P.K., Criscione J.D., Liu X., Beutner G. (2022). Uncompensated mitochondrial oxidative stress underlies heart failure in an iPSC-derived model of congenital heart disease. Cell Stem Cell.

[47] Kowalczyk W., Romanelli L., Atkins M., Hillen H., González-Blas C.B., Jacobs J., Xie J., Soheily S., Verboven E., Moya I.M. (2022). Hippo signaling instructs ectopic but not normal organ growth. Science.

[48] Saunders A., Huang X., Fidalgo M., Reimer M.H., Faiola F., Ding J., Sánchez-Priego C., Guallar D., Sáenz C., Li D. (2017). The SIN3A/HDAC Corepressor Complex Functionally Cooperates with NANOG to Promote Pluripotency. Cell Rep..

[49] van Oevelen C., Bowman C., Pellegrino J., Asp P., Cheng J., Parisi F., Micsinai M., Kluger Y., Chu A., Blais A. (2010). The Mammalian Sin3 Proteins Are Required for Muscle Development and Sarcomere Specification. Mol. Cell. Biol..

[50] Gao Z., Ure K., Ding P., Nashaat M., Yuan L., Ma J., Hammer R.E., Hsieh J. (2011). The Master Negative Regulator REST/NRSF Controls Adult Neurogenesis by Restraining the Neurogenic Program in Quiescent Stem Cells. J. Neurosci..

[51] Witteveen J.S., Willemsen M.H., Dombroski T.C.D., Van Bakel N.H.M., Nillesen W.M., Van Hulten J.A., Jansen E.J.R., Verkaik D., Veenstra-Knol H.E., Van Ravenswaaij-Arts C.M.A. (2016). Haploinsufficiency of MeCP2-interacting transcriptional co-repressor SIN3A causes mild intellectual disability by affecting the development of cortical integrity. Nat. Genet..

[52] Ota M., Sasaki H. (2008). Mammalian Tead proteins regulate cell proliferation and contact inhibition as transcriptional mediators of Hippo signaling. Development.

[53] Xin M., Kim Y., Sutherland L.B., Qi X., McAnally J., Schwartz R.J., Richardson J.A., Bassel-Duby R., Olson E.N. (2011). Regulation of Insulin-Like Growth Factor Signaling by Yap Governs Cardiomyocyte Proliferation and Embryonic Heart Size. Sci. Signal..

[54] Zhu F., Zhu Q., Ye D., Zhang Q., Yang Y., Guo X., Liu Z., Jiapaer Z., Wan X., Wang G. (2018). Sin3a–Tet1 interaction activates gene transcription and is required for embryonic stem cell pluripotency. Nucleic Acids Res..

[55] Peinado H., Ballestar E., Esteller M., Cano A. (2004). Snail Mediates E-Cadherin Repression by the Recruitment of the Sin3A/Histone Deacetylase 1 (HDAC1)/HDAC2 Complex. Mol. Cell. Biol..

[56] Ocaña O.H., Coskun H., Minguillón C., Murawala P., Tanaka E.M., Galceran J., Muñoz-Chápuli R., Nieto M.A. (2017). A right-handed signalling pathway drives heart looping in vertebrates. Nature.

[57] Murray S.A., Gridley T. (2006). Snail family genes are required for left–right asymmetry determination, but not neural crest formation, in mice. Proc. Natl. Acad. Sci. USA.

[58] Logan M., Pagán-Westphal S.M., Smith D.M., Paganessi L., Tabin C.J. (1998). The Transcription Factor Pitx2 Mediates Situs-Specific Morphogenesis in Response to Left-Right Asymmetric Signals. Cell.

[59] Yoshioka H., Meno C., Koshiba K., Sugihara M., Itoh H., Ishimaru Y., Inoue T., Ohuchi H., Semina E.V., Murray J.C. (1998). Pitx2, a Bicoid-Type Homeobox Gene, Is Involved in a Lefty-Signaling Pathway in Determination of Left-Right Asymmetry. Cell.

[60] Nomura M., Li E. (1998). Smad2 role in mesoderm formation, left–right patterning and craniofacial development. Nature.

[61] Gilljam T., McCrindle B.W., Smallhorn J.F., Williams W.G., Freedom R.M. (2000). Outcomes of left atrial isomerism over a 28-year period at a single institution. J. Am. Coll. Cardiol..

